# A Developmental Taxonomy of Autism and the Precision Timing of the Cheung Glutamatergic Regimen

**DOI:** 10.7759/cureus.107120

**Published:** 2026-04-15

**Authors:** Ngo Cheung

**Affiliations:** 1 Psychiatry, Cheung Ngo Medical Limited, Hong Kong, HKG

**Keywords:** autism, glutamatergic, lrrk2, precision medicine, pruning

## Abstract

Autism spectrum disorder is far more heterogeneous than traditionally assumed, with age at diagnosis reflecting distinct polygenic factors and fundamentally different developmental trajectories rather than simple differences in detection. What was previously described as a single two-stage synaptic course--early overgrowth followed by later depletion--now appears as two mechanistically separable subtypes. Early-diagnosed autism is characterised by a "Failure to Build," driven by deficits in mitochondrial function, ribosomal biogenesis, and cytoskeletal integrity during initial synapse formation. In contrast, late-diagnosed autism reflects a "Failure to Refine," arising from astrocyte dysfunction, complement-mediated over-pruning, and LRRK2 dysregulation during adolescent circuit remodelling, with strong transcriptomic convergence to ADHD.

Against this backdrop, the Cheung glutamatergic regimen--a low-cost, orally available combination of dextromethorphan, a CYP2D6-inhibiting antidepressant, piracetam, and L-glutamine--is proposed as a ketamine-mimetic plasticity agent. The regimen is contraindicated in the early-diagnosed ("Build") subtype and in all children under approximately 10 years due to the risk of exacerbating bioenergetic stress and excitotoxicity. However, it may prove restorative in post-pubertal individuals with late-diagnosed autism and ADHD comorbidity by counteracting astrocyte dysfunction, excessive complement tagging, and LRRK2-related pruning dysregulation.

This refined, subtype- and timing-specific framework carries important clinical implications and underscores the urgent need for biomarker-stratified randomised trials using synaptic density imaging and circuit-level outcomes.

Keywords: autism heterogeneity, synaptic pruning, LRRK2, transcriptome-wide association study, glutamatergic enhancement, precision medicine

## Introduction and background

Heterogeneity in autism and the significance of diagnostic timing

Autism spectrum disorder has been recognised as a remarkably varied condition for some time, yet it is only in recent years that investigators have started to grasp how much the age at which a person is diagnosed may point to genuinely different biological underpinnings rather than mere differences in service access or clinical awareness. For much of the field's history, the dominant assumption was that the core features of autism appear early and stay more or less constant across the lifespan [1]. Epidemiological findings have complicated this picture, however, because a sizeable and increasing share of individuals do not receive a diagnosis until middle childhood, the teenage years, or even adulthood [1,2]. This pattern has pushed researchers toward thinking of autism less as a fixed entity and more as a developmental process whose outward presentation can follow quite different courses over time [1-3].

Polygenic evidence for separable early- and late-diagnosed trajectories

A pivotal contribution by Zhang et al. [3], drawing on longitudinal data from four independent birth cohorts, offered the first rigorous demonstration that age at diagnosis carries biological meaning beyond the simple question of when someone happens to be assessed. Their analyses uncovered two partly distinct polygenic factors whose genetic correlation with one another is only moderate (rg = 0.38). The factor tied to early diagnosis maps onto marked social-communication difficulties that are already visible during the preschool period. The factor tied to later diagnosis, by contrast, is characterised by relatively intact early functioning that gives way to a steep increase in socioemotional and behavioural problems during late childhood and adolescence [3]. The two factors also part company in their genetic links to other psychiatric conditions; the late-diagnosed trajectory shares considerably more genetic overlap with ADHD, depression, and anxiety than its early-diagnosed counterpart [3].

Transcriptomic pathways: build versus refine

Work at the transcriptomic level has started to flesh out how these polygenic differences get translated into divergent neurodevelopmental outcomes [4,5]. Transcriptome-wide association studies of early-diagnosed autism point to enrichment of pathways governing mitochondrial oxidative phosphorylation, ribosomal biogenesis, cytoskeletal dynamics, and cellular senescence--all of which are central to the initial formation and stabilisation of synapses during fetal and early postnatal life [4]. Late-diagnosed autism, on the other hand, shows a distinct transcriptomic signature weighted toward adult astrocyte markers, complement cascade components, genes involved in synapse pruning, and LRRK2 expression within limbic circuits [4,5]. This contrast has been framed as a "build versus refine" distinction: early-diagnosed autism appears to involve a fundamental failure in laying down synaptic networks, whereas late-diagnosed autism--along with much of its comorbidity with ADHD--seems to stem from disordered pruning and circuit remodelling during adolescence [4,5].

Convergence with ADHD and synaptic imaging findings

Supporting this framework, parallel transcriptome-wide analyses of ADHD summary statistics have shown that the transcriptomic profile of ADHD aligns more than three times as strongly with the late-diagnosed autism pattern as with the early-diagnosed form [5]. This fits with longstanding clinical observations and the elevated rates of co-occurring ADHD among those diagnosed with autism later in life [6,7]. The build-versus-refine model also helps make sense of what had seemed like contradictory post-mortem and neuroimaging data: some autistic brains display an early surplus of synapses coupled with inadequate pruning [8], while others show net synaptic loss by the time the person reaches adulthood [9].

A subtype-informed therapeutic rationale

With these subtype-specific synaptic trajectories as context, the present review considers whether the Cheung glutamatergic regimen--a low-cost, fully oral combination of dextromethorphan (acting as an NMDA receptor blocker), a CYP2D6-inhibiting antidepressant, piracetam (an AMPA receptor modulator), and L-glutamine--could serve as a plasticity-enhancing intervention comparable in mechanism to ketamine [10]. The rationale is that by producing a controlled surge in glutamate while favouring AMPA-mediated transmission and maintaining presynaptic glutamine stores, the regimen may promote BDNF release and mTOR-dependent synaptogenesis [11,12]. If diagnostic timing tells us something real about which synaptic processes have gone awry, then matching the intervention to the subtype becomes just as important as getting the timing right.

## Review

Divergent synaptic life-cycles in autism: a build-versus-refine taxonomy

For a long time, the field has worked with a fairly straightforward story about how synapses go wrong in autism: connections are overproduced early on, pruning lags behind, and at some later point, there appears to be a compensatory loss. That account was not invented out of thin air--it captured something real--but it papered over meaningful differences in when and how synaptic development actually derails. Longitudinal cohort data paired with genomic structural equation modelling have since demonstrated that the age at which a person is diagnosed tracks two polygenic factors that are only modestly correlated with one another (rg = 0.38) and that follow distinct clinical courses [3]. One group already shows elevated difficulties from the preschool years onward; the other looks relatively typical early in life before experiencing a steep climb in socioemotional problems during late childhood and adolescence. Transcriptome-wide association studies have filled in the mechanistic gap by uncovering two separable phases in the synaptic life-cycle--what can usefully be called the "build" and "refine" stages [4,5]. Together, these findings change how we should think about when synaptic surplus or depletion is actually happening and, just as importantly, when glutamatergic interventions might do good versus when they risk doing harm.

Prenatal to early childhood: the "build" phase (early-diagnosed subtype)

The stretch from the prenatal period through roughly the first two years after birth is consumed by rapid synaptogenesis--a process that places enormous demands on the cell's capacity to generate energy, synthesise proteins, and remodel its internal skeleton. In the early-diagnosed subtype, the genes whose expression is most strongly implicated are those encoding mitochondrial oxidative phosphorylation subunits (COX5A and several NDUFA components of Complex I stand out), ribosomal proteins, microtubule-associated proteins like MAP1A, and regulators tied to cellular senescence and HLA-mediated immune signalling [4]. These are exactly the pathways the brain leans on most heavily when it is assembling and stabilising new synapses at speed. If they are disrupted, the outcome is not the classic overabundance of dendritic spines that was long assumed to be universal in autism. Instead, what appears to go wrong is something more basic: a failure to build and hold together synaptic networks from the very start [4,13]. The expected phenotype is a globally reduced synaptic density that is already detectable in early childhood, with particular impairment in long-range connectivity across hippocampal and frontal circuits [4].

This reframing sheds light on some apparent contradictions in older work. Accelerated head growth and macrocephaly have been documented in a notable subset of autistic infants [14,15], and those findings were often taken as evidence of widespread synaptic excess. The transcriptomic picture of the early-diagnosed group, however, suggests that such overgrowth may be less universal than previously supposed. It could instead reflect secondary compensatory or inflammatory processes rather than a genuine surfeit of synapses. Studies examining brain tissue after death and brain imaging that separate participants based on when they were first diagnosed would be necessary to rigorously evaluate this prediction. That said, the molecular data already challenge the "overcrowded brain" storyline that has dominated so much of the conversation around early intervention [13,16].

**Figure 1:**
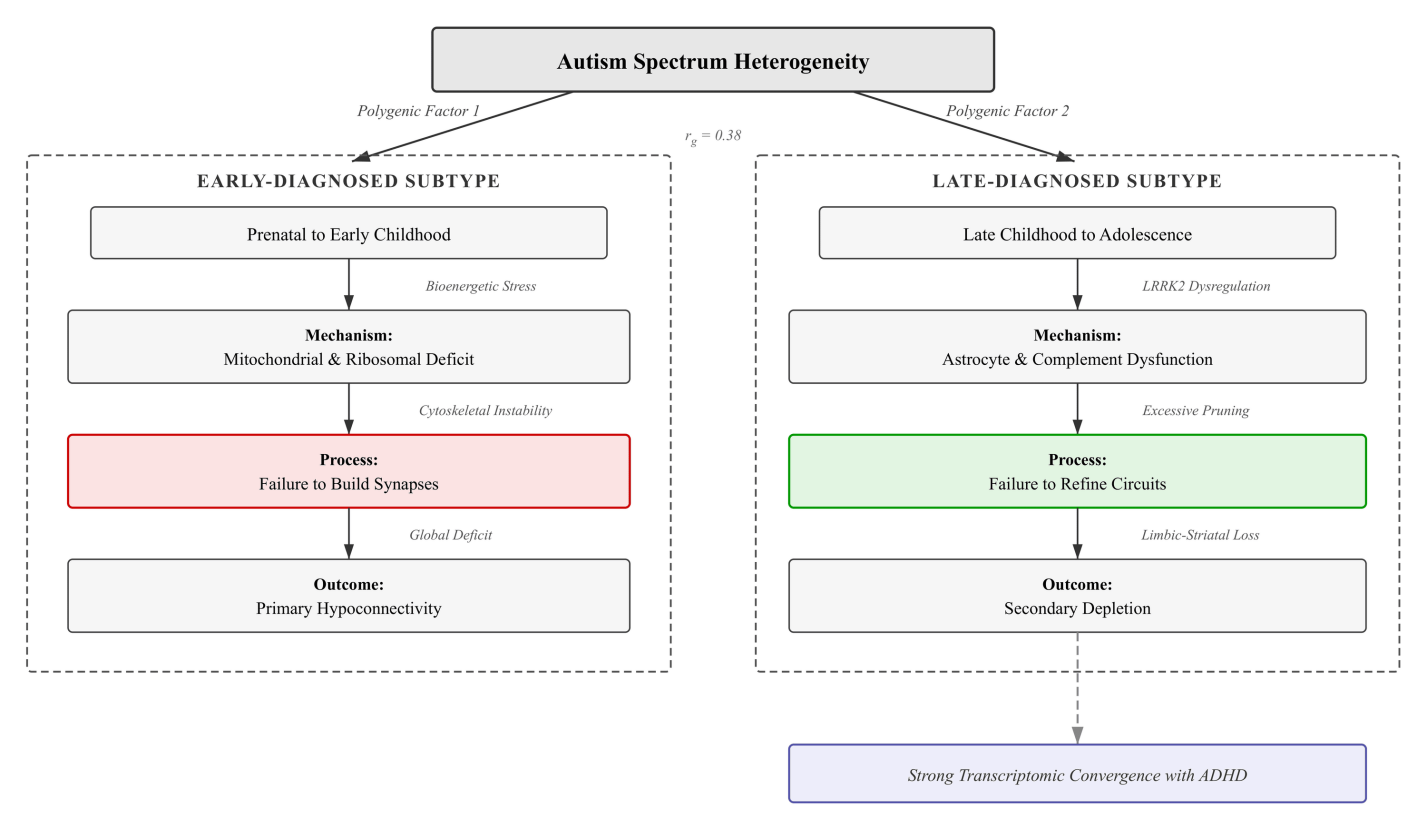
Divergent Synaptic Life-Cycles in Autism The taxonomy separates the condition into two mechanistically distinct pathways. The Early-Diagnosed trajectory (left) is characterised by a "Failure to Build," driven by mitochondrial and ribosomal deficits during the initial formation of synapses. The Late-Diagnosed trajectory (right) is characterised by a "Failure to Refine," where initial development is largely intact, but adolescent circuit remodelling is disrupted by astrocyte dysfunction, complement-mediated over-pruning, and LRRK2 dysregulation. This latter pathway shows significant biological overlap with ADHD. Credits to Ngo Cheung.

See Figure 1 for a timeline contrasting neurotypical and early-diagnosed "Build Failure" trajectories.

Late childhood to adolescence: the "refine" phase (late-diagnosed subtype)

By late childhood, the brain's main developmental task shifts from construction to sculpting. Experience-dependent pruning and circuit refinement take over as the dominant processes, especially within prefrontal, limbic, and striatal networks. In the late-diagnosed subtype, the strongest transcriptomic signals point to adult astrocyte marker genes, complement cascade components, synapse-pruning regulators, and--most strikingly--LRRK2, which emerged as the single strongest hit across the entire analysis [4]. These signals cluster in brain structures that handle social and reward-related processing, particularly the amygdala and nucleus accumbens, which fits well with the adolescent surge in social rigidity and emotional dysregulation that marks this trajectory [3,4].

The developmental sequence here is essentially the reverse of what happens in the early-diagnosed group: synaptic density starts out normal or close to it, but pruning during adolescence becomes either too aggressive or poorly timed. The result is the "catch-up" cortical thinning that longitudinal MRI studies have picked up, culminating in the roughly 17% reduction in synaptic density that PET imaging has documented in autistic adults [9]. These same mechanisms go a long way toward explaining why the late-diagnosed subtype overlaps so heavily with ADHD at both the clinical and transcriptomic levels. When ADHD summary statistics were run through parallel analyses, the resulting profiles correlated more than three times as strongly with the late-diagnosed autism arm as with the early-diagnosed form (Pearson r = 0.310 versus 0.094). The two conditions share negative LRRK2 directionality, astrocyte and complement signals, and enrichment for pruning-related pathways, though ADHD adds its own distinctive accents in long-term potentiation and monoaminergic signalling [5,6].

See Figure 1 for the contrasting "Refine Failure" timeline and ADHD convergence.

Adulthood: the inversion and subtype divergence

By the time both groups reach adulthood, they have arrived at a broadly similar endpoint--under-connectivity--but they got there by different routes. The late-diagnosed arm accounts for much of the net synaptic depletion observed in adults, driven by excessive elimination during adolescence and poor maintenance of long-range limbic-prefrontal circuits. The early-diagnosed arm, having started from a position of inadequate synapse building, shows persistently low synaptic density without that dramatic adolescent drop-off. This divergence in trajectories goes some way toward explaining why findings of adult synaptic loss [9] and the patterns of psychiatric comorbidity that tend to accompany them line up much more closely with individuals who were diagnosed later in life [3,7]. It also highlights a practical problem with older assumptions: talking about "excess synapses in autism" as though it were a universal feature has limited value when the underlying biology is not the same across individuals [3,4]. The interventions we design should not be uniform either.

**Figure 2:**
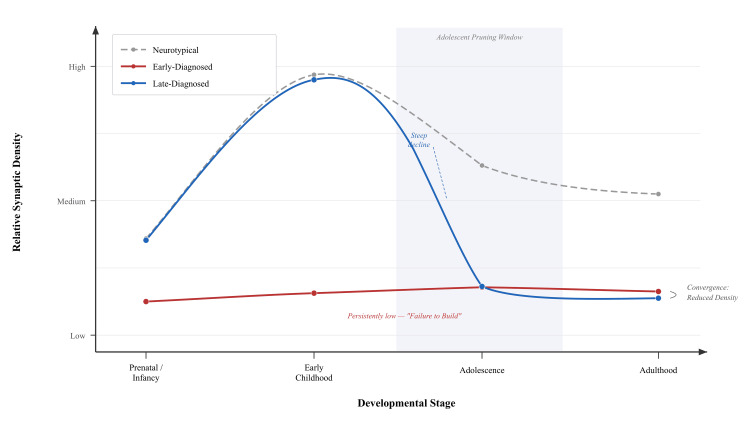
Trajectory Inversion and Convergence. Trajectory Inversion and Convergence. Schematic representation of synaptic density across developmental stages. The Early-Diagnosed group (red) starts with a deficit and maintains a persistently low baseline throughout life. The Late-Diagnosed group (blue) begins with near-normal density, closely tracking the neurotypical reference (dashed grey), but undergoes a precipitous decline during the adolescent pruning phase (shaded region). While both groups converge at reduced synaptic density in adulthood, the developmental route to this endpoint differs fundamentally, dictating distinct therapeutic requirements. Credits to Ngo Cheung.

See Figure 2 for split-panel adult trajectories illustrating subtype-specific inversion.

Pulling these strands together, what emerges is not a single story of synaptic dysregulation but a developmental taxonomy with two arms. That taxonomy has direct implications for deciding when plasticity-enhancing strategies are likely to be safe and when they risk being counterproductive.

Contraindication in early childhood: now subtype-stratified

The developmental taxonomy outlined above demands that we revisit the safety question with sharper tools than before. What used to look like a single, blanket "danger zone" covering all of early childhood now breaks apart along biological lines that are specific to each subtype.

The Early-Diagnosed ("Build") Subtype

In children whose profile fits the early-diagnosed arm, the brain is already contending with foundational shortfalls in mitochondrial energy production, ribosomal protein synthesis, and the cytoskeletal assembly work that underpins synapse formation [4,13,16]. Neurons in this group are operating under chronic energy strain, and there are early molecular signs of senescence-related stress on top of that. Introducing an intervention that ramps up glutamatergic signalling--the sequence of transient NMDA blockade, a resulting glutamate surge, AMPA facilitation, and downstream mTOR activation--is not just a matter of adding fuel to an already hot fire. The real danger is that energy-starved cells, already tilting toward senescence, get pushed into outright excitotoxic damage [11,17,18]. The very mechanisms that could make the Cheung regimen restorative at a later stage of life become actively destructive when the cellular hardware needed to cope with the resulting calcium influx and metabolic load is already running on fumes.

The Late-Diagnosed ("Refine") Subtype in Childhood

For children who will eventually fit the late-diagnosed pattern, the picture is less clear-cut. Their early synaptic density is not yet excessive, and the vulnerabilities that define their trajectory--astrocyte dysfunction, complement-driven pruning--do not really come online until around ages nine to eleven, when the adolescent refinement phase kicks in [3,4]. Caution remains essential through puberty, but the window of greatest risk is narrower, and the biological reasons behind it are different from those in the early-diagnosed group [3,4]. Even so, any strategy that boosts glutamate signalling before that developmental transition has the potential to disrupt normal early synaptic stabilisation, and it should be treated with considerable care [11,17,18].

An Updated Clinical Stance

These considerations point toward a straightforward position: the Cheung glutamatergic regimen--dextromethorphan combined with a CYP2D6 inhibitor, piracetam, and L-glutamine--is contraindicated in all children before puberty and in any individual whose clinical and biological profile matches the early-diagnosed trajectory, no matter what their chronological age happens to be. The combination should not enter the conversation until well after puberty, and only then for people who clearly show a late-emergent, "Refine"-type phenotype. This is not a counsel of despair but rather a call for precision. The same regimen that could worsen core difficulties in a young child whose neurons are bioenergetically vulnerable may later turn out to be a well-targeted tool for circuit restoration, once the developmental window has moved on and the brain's needs have fundamentally changed.

**Figure 3:**
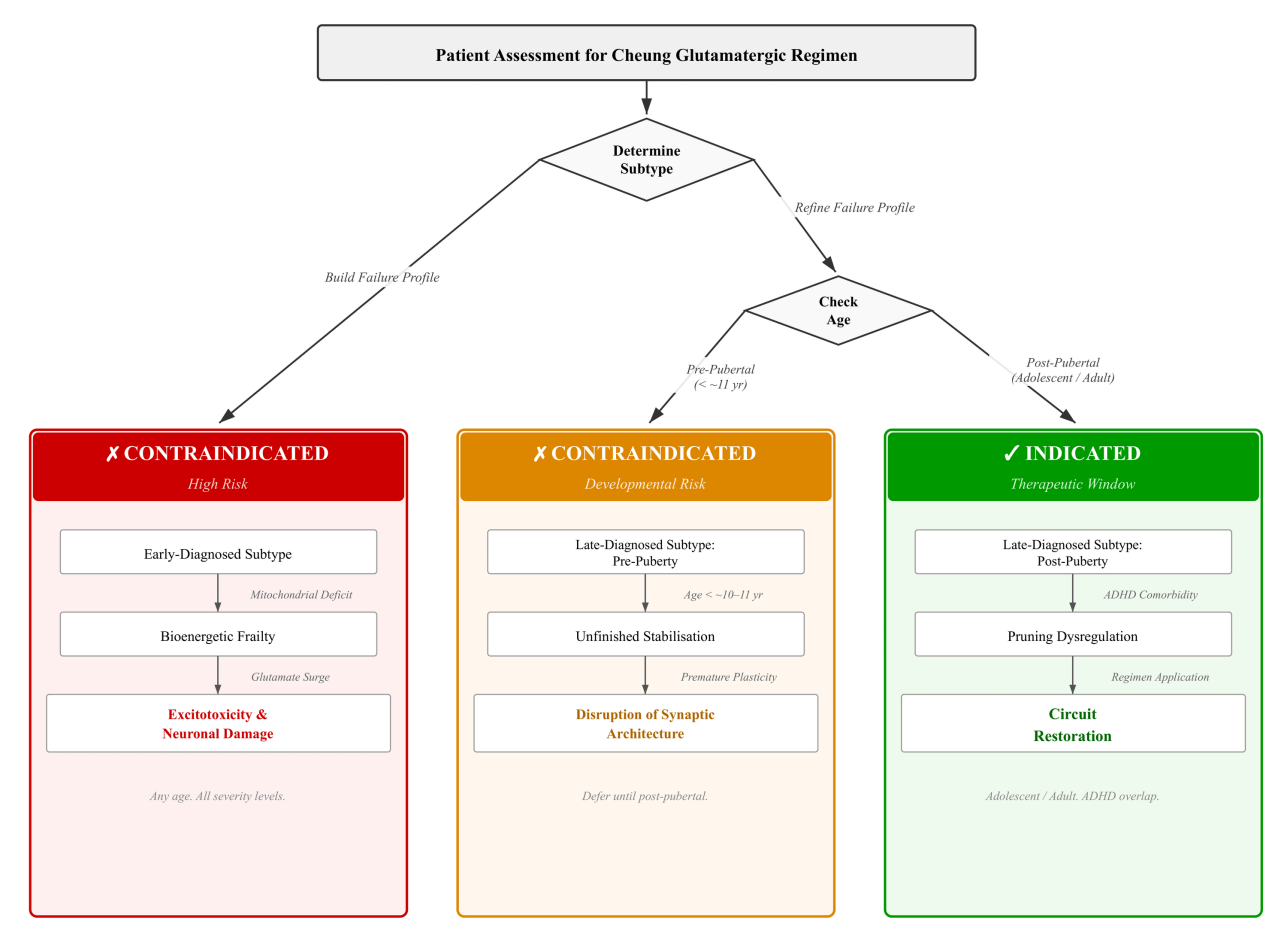
The Subtype-Stratified Safety Framework The Cheung glutamatergic regimen is strictly contraindicated in the Early-Diagnosed "Build" subtype (red) due to the risk of excitotoxicity in energy-starved neurons. It is also contraindicated in Late-Diagnosed children prior to puberty (amber) to avoid disrupting natural synaptic stabilisation. The therapeutic window opens only in the Late-Diagnosed "Refine" subtype during adolescence and adulthood (green), where the goal is to counteract excessive pruning. Credits to Ngo Cheung.

See Figure 3 for the subtype-stratified Danger Zone illustration.

Therapeutic potential in adolescence and beyond: precision targeting of the "refine" deficit

Once the brain has moved past the early construction phase, the therapeutic calculus changes in an important way. For people on the late-diagnosed trajectory--and especially those who also meet criteria for ADHD--the stretch from mid-adolescence onward may represent the most promising window for trying the Cheung glutamatergic regimen. In this "Refine" subtype, the central problem is not that too few synapses were built in the first place but that pruning has gone wrong: it is mistimed or overshoots, driven by astrocyte dysfunction, overactive complement signalling, and LRRK2 dysregulation concentrated in limbic and striatal circuits [4]. The same oral combination that poses real risks to bioenergetically fragile young children may, in this older group, help restore a measure of balance after adolescent circuit sculpting has cut too deep.

Mechanistic Fit

The alignment between what the regimen does and what this subtype needs is worth spelling out. The controlled glutamate surge--set in motion by transient NMDA blockade and carried forward through AMPA receptors--triggers BDNF release and a degree of mTOR engagement, both of which are known to support the stabilisation of existing synapses [11,12]. In the late-diagnosed context, this cascade could help through at least four routes that work together rather than in isolation.

First, glutamine supplementation may shore up the astrocyte side of glutamate buffering and lactate shuttling, counteracting metabolic strain that in this subtype appears to be a secondary rather than a primary problem [19,20]. Second, the plasticity signals the regimen generates may reduce the excessive complement tagging of synapses that ought to be kept rather than eliminated [21]. Third, the intervention could help normalise how LRRK2 regulates synaptic vesicle trafficking and autophagy--pathways that have come up repeatedly in both autism and ADHD research [22,23]. Fourth, it may rebalance long-term potentiation within limbic-striatal circuits, addressing the distinctive monoaminergic flavour that characterises cases where ADHD and late-diagnosed autism overlap [5].

If these mechanisms hold up, the expected clinical gains would include stronger long-range cortico-cortical and prefrontal-limbic connectivity [11,12], improved gamma-band synchrony of the kind that matters for social cognition [24,25], and measurable improvements in social reciprocity and executive function [7,26].

Possible Synergy With LRRK2 Kinase Inhibitors

One especially interesting possibility is combining the glutamatergic regimen with the LRRK2 kinase inhibitors now making their way through clinical trials. Several candidates--including BIIB122 (DNL151), being tested in the ongoing LUMA trial, and NEU-411 in the NEULARK Phase 2 study--have shown encouraging target engagement and acceptable safety profiles in early Parkinson's disease programmes [27,28]. Because the transcriptome-wide data show reduced LRRK2 expression in the late-diagnosed arm [4], a combined strategy that uses the glutamatergic regimen to drive plasticity while separately fine-tuning LRRK2 activity could offer more precise circuit repair than either approach on its own [22,23,27,28].

Why This Subgroup is a Good Candidate

From a practical standpoint, this particular clinical group is well-suited to a low-cost oral intervention. ADHD co-occurs at rates three to four times higher in late-diagnosed than in early-diagnosed autism, the sex ratio moves closer to even, and many of these individuals only come to clinical attention during the adolescent rise in socioemotional difficulties [7]. For patients in this position, an accessible regimen that can be started and adjusted in ordinary outpatient settings could fill a treatment gap that currently has very little in it.

**Figure 4:**
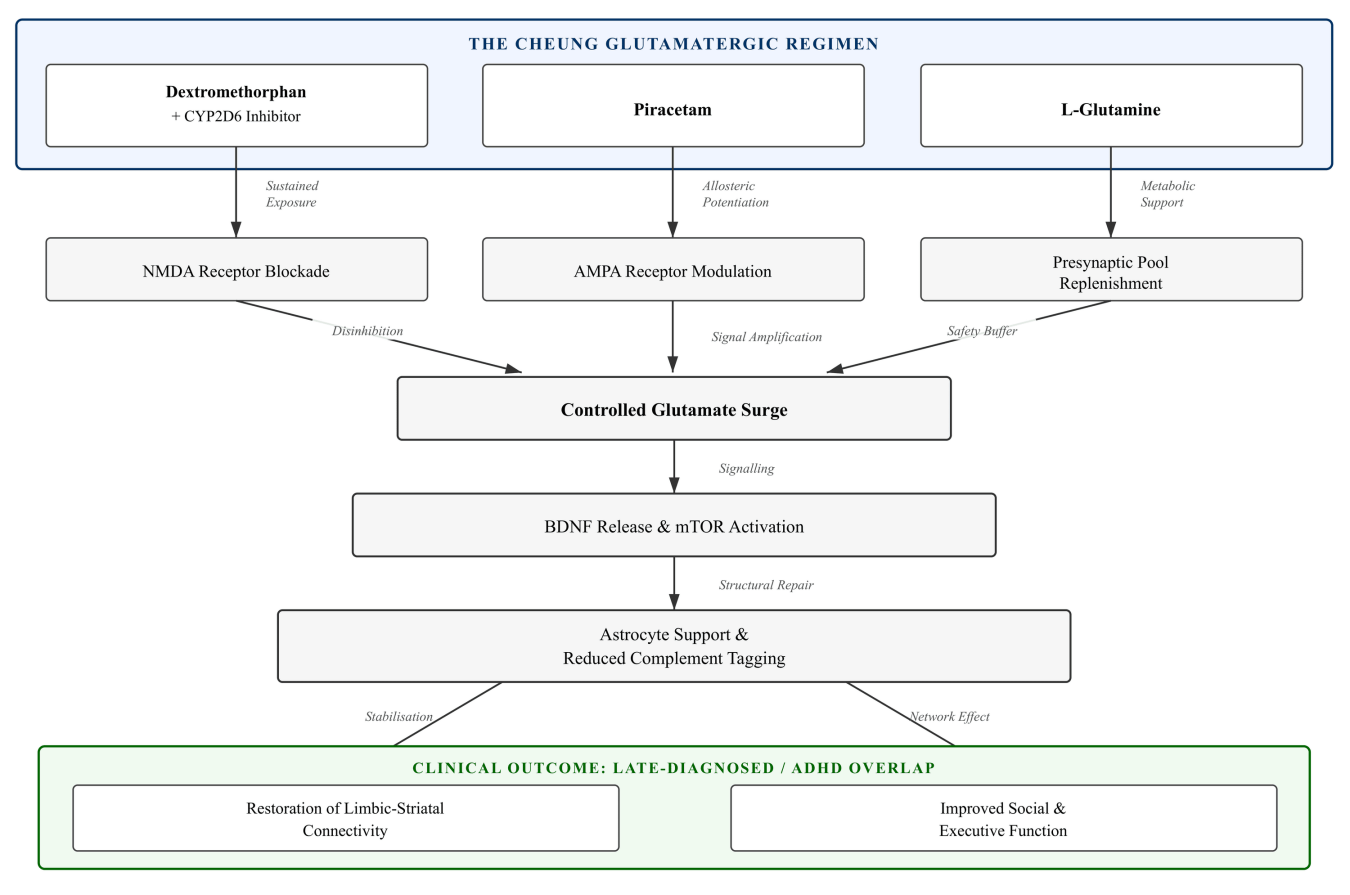
Therapeutic Mechanism in the "Refine" Subtype The regimen combines three pharmacological vectors: (1) Dextromethorphan (boosted by CYP2D6 inhibition) blocks NMDA receptors to trigger a glutamate surge; (2) Piracetam sensitises AMPA receptors to capture this signal; (3) L-Glutamine supports astrocyte metabolism and prevents depletion. In the Late-Diagnosed brain, this cascade promotes BDNF/mTOR activity, counteracts excessive complement-mediated pruning, and restores connectivity in limbic-striatal circuits. Credits to Ngo Cheung.

See Figure 4 for the updated Therapeutic Window highlighting the late-diagnosed and ADHD-overlap pathway.

Implications for precision medicine and future directions

Accepting that autism involves at least two biologically separable synaptic life-cycles changes the therapeutic conversation in a fundamental way. It is no longer enough to ask whether a given intervention "works for autism." The prior question--which autism, and at what point in development--has to come first. Getting the stratification right is therefore not a theoretical nicety but the practical foundation for safer and more effective care.

Stratification Tools Already Within Reach

Several approaches to subtyping are available now or close to it. Age at diagnosis is the simplest and most immediately usable proxy: children diagnosed before roughly nine to eleven years tend to fall on the early "Build" trajectory, while those diagnosed later cluster with the "Refine" arm [3]. Where resources allow, polygenic factor scores calculated from the age-stratified genome-wide association data can tighten this classification further. Beyond genomics, a number of emerging biomarkers promise even finer resolution. Circulating complement fragments such as C1q and C3 offer a window onto pruning activity [21,29]. Peripheral readouts of LRRK2 pathway function may flag limbic vulnerability. And resting-state EEG gamma-band power or synchrony provides a non-invasive measure of long-range circuit integrity--something already known to be disrupted in the late-diagnosed group [24,26]. These markers still need validation in prospective cohorts, but once that work is done, there is no reason they could not move into routine clinical use.

Subtype-Specific Treatment Pathways

With stratification in hand, treatment pathways should diverge. For individuals on the early-diagnosed arm, the emphasis remains on early intensive behavioural intervention alongside strategies that support mitochondrial function and cellular energy supply. Recent double-blind trials of targeted mitochondrial supplements have been encouraging, with improvements reported in social withdrawal, hyperactivity, and markers of mitochondrial activity itself [13,16,30]. Glutamatergic enhancement has no place in this group.

The picture flips for post-pubertal individuals on the late-diagnosed trajectory, particularly those with prominent ADHD comorbidity. These are the clearest candidates for the Cheung glutamatergic regimen. Use could reasonably begin in mid-adolescence, and ideally it would be titrated alongside the LRRK2 kinase inhibitors that are already advancing through Parkinson's disease trials, and that may normalise the very pruning dysregulation the transcriptome-wide data have highlighted [27,31]. Pairing plasticity enhancement with pruning modulation in this way could address the adolescent decompensation that is so common in this subgroup.

Designing the Next Generation of Trials

The logical next step is biomarker-stratified randomised controlled trials. Primary endpoints should include objective measures of synaptic density using ¹¹C-UCB-J PET, limbic-prefrontal connectivity assessed with functional MRI, and EEG gamma-band metrics. Secondary outcomes would track social reciprocity, executive function, and adaptive behaviour. Pre-specified subgroup analyses by diagnostic timing and polygenic loading should be built in from the start. Trials designed this way would do double duty, testing the regimen and putting the build-versus-refine model itself to a direct empirical test.

Broader Relevance Across Psychiatry

Finally, the same developmental taxonomy may shed light on other conditions where the age at which problems first appear varies strikingly from person to person. Parallel transcriptomic and longitudinal approaches could prove valuable in ADHD, in certain presentations of obsessive-compulsive disorder, and in late-adolescent forms of schizophrenia--all conditions where excessive or poorly timed synaptic pruning has been implicated [6,32,33]. In this sense, the framework sketched here is not just about autism. It offers a broader roadmap for precision psychiatry across the neurodevelopmental and psychiatric spectra.

## Conclusions

Autism spectrum disorder is not one static condition but a family of related neurodevelopmental trajectories that part company at the level of when and how synaptic development goes wrong. Early-diagnosed autism is marked by foundational shortfalls in mitochondrial function, ribosomal biogenesis, and cytoskeletal integrity--problems that impair the initial construction of synaptic networks. Late-diagnosed autism, along with its frequent overlap with ADHD, arises instead from dysregulated astrocyte function, complement-driven pruning, and LRRK2 signalling during the adolescent refinement phase. These distinct biological windows carry direct and different implications for intervention.

The Cheung glutamatergic regimen holds promise as a low-cost plasticity enhancer, but it carries real risk of worsening neuronal stress in the early "Build" subtype and in all pre-pubertal children. Its greatest therapeutic potential lies elsewhere: in post-pubertal individuals on the late-diagnosed trajectory, where it may help counteract excessive pruning, restore astrocyte support, and rebalance limbic circuits. This regimen is not a one-size-fits-all treatment. It is a timing- and subtype-specific precision tool, and its greatest promise sits squarely in the adolescent and adult "Refine" arm of autism.
